# Metabolome of Cerebral Thrombi Reveals an Association between High Glycemia at Stroke Onset and Good Clinical Outcome

**DOI:** 10.3390/metabo10120483

**Published:** 2020-11-25

**Authors:** Laurent Suissa, Jean-Marie Guigonis, Fanny Graslin, Emilie Doche, Ophélie Osman, Yves Chau, Jacques Sedat, Sabine Lindenthal, Thierry Pourcher

**Affiliations:** 1 Laboratory Transporter in Imaging and Radiotherapy in Oncology (TIRO), Direction de la Recherche Fondamentale (DRF), Institut des Sciences du Vivant Fréderic Joliot, Commissariat à l′Energie Atomique et aux Énergies Alternatives (CEA), University Côte d’Azur, F-06107 Nice, France; laurent.suissa@ap-hm.fr (L.S.); Jean-Marie.GUIGONIS@univ-cotedazur.fr (J.-M.G.); fanny.graslin@univ-cotedazur.fr (F.G.); sabine.lindenthal@univ-cotedazur.fr (S.L.); 2 Stroke Unit, University Hospital, F-13385 Marseille, France; emilie.doche@ap-hm.fr (E.D.); ophelie.osman@ap-hm.fr (O.O.); 3 Interventional Radiology Unit, University Hospital, F-06001 Nice, France; chau.hdy@chu-nice.fr (Y.C.); sedat.j@chu-nice.fr (J.S.)

**Keywords:** stroke, thrombectomy, thrombosis, metabolomics, glucose, sorbitol

## Abstract

Despite the fact that glucose is the main fuel of the brain, hyperglycemia at hospital admission is generally associated with a poor functional outcome in stroke patients. This paradox may be explained by the lack of information about the blood glucose level at stroke onset. Here, we analyzed the metabolome of blood cells entrapped in cerebral thrombi to gain insight into their metabolism at stroke onset. Fourty-one consecutive stroke patients completely recanalized by mechanical thrombectomy within 6 h were included. The metabolome of retrieved thrombi was analyzed by liquid chromatography tandem with mass spectrometry. Discriminant Analysis (sparse Partial Least Squares Discriminant Analysis (sPLS-DA)) was performed to identify classification models and significant associated features of favorable clinical outcome at 3 months (modified Rankin Scale (mRS) < 2). sPLS-DA of the metabolomes of cerebral thrombi discriminated between stroke patients with a favorable or poor clinical outcome (Area Under the Curve (AUC) = 0.992 (0.931–1)). In addition, our results revealed that high sorbitol and glucose levels in the thrombi positively correlated with favorable clinical outcomes. Sorbitol, a short-term glycemic index reflecting a high blood glucose level at stroke onset, was found to be an independent predictor of good outcome (AUC = 0.908 (0.807–0.995)). This study demonstrates that a high blood glucose level at stroke onset is beneficial to the clinical outcome of the patient.

## 1. Introduction

In the last decade, remarkable progress into reperfusion treatment of patients with acute stroke with large artery occlusion has led to improved clinical outcomes [1]. Endovascular thrombectomy after large vessel ischemic stroke within 6 h of symptom onset is proposed in addition to intravenous thrombolysis by alteplase, if eligible [2]. Despite the unquestionable value of this major advance, only less than half of the treated patients show clinical benefit from recanalization leading to the common assumption that recanalization is considered as necessary but not sufficient [1,3].

Admission hyperglycemia has been strongly associated with a poor functional outcome in patients treated with intravenous recombinant tissue Plasminogen Activator (rt-PA) and more recently, in stroke patients with large vessel occlusion treated with mechanical thrombectomy [4,5]. However, the molecular mechanisms underlying the robust association between admission hyperglycemia and poor clinical outcome are still uncertain. The blood glucose level is certainly a major parameter for the prediction of clinical stroke outcome but measurement of blood glucose at admission, before reperfusion, is affected by a stress response to acute neurologic injury [6,7] and does not automatically reveal the glycemia at stroke onset, which remains unknown in clinical practice.

According to the review of Robbins and Swanson [6], glucose has dual opposing effects on stroke and on the reperfusion setting. While the deleterious role of glucose on reperfusion is supported by experimental and clinical data, the beneficial effect of glucose could theoretically be supported by its capacity to produce energy (ATP) even in the absence of oxygen (anaerobic glycolysis) in the ischemic setting [6]. Indeed, some observations reviewed by Robbins and Swanson argue for a beneficial effect of glucose on stroke, which has not been demonstrated in clinical practice until now because glycemia at stroke onset remains unknown [6]. New insight into this area is needed for target neuroprotection strategies for recanalized stroke patients.

Metabolomic analyses using a mass spectrometer allow the study of many substrates, intermediates and products of cell metabolism. However, to our knowledge, the metabolome of cerebral thrombi has not yet been studied. Here, we hypothesized that the analysis of the cells entrapped in the cerebral thrombi retrieved during the acute phase of stroke patients could provide information about glucose metabolism of blood cells at stroke onset.

## 2. Results

### 2.1. Patient Characteristics

Among the 59 stroke patients included in the ThrombiOMIC database, 41 patients met the inclusion criteria for our study. The baseline characteristics of these 41 stroke patients are shown in Table 1. All patients (age: 74.8 ± 13.2 years, men: 17/41 (41.5%), baseline National Institutes of Health Stroke Scale (NIHSS): 17 (15–22)) were successfully recanalized (mTICI 2b/3) by mechanical thrombectomy for a middle cerebral artery occlusion within 6 h from stroke onset (time elapsed from stroke onset to recanalization: 252.4 ± 62.2 min) according to American Heart Association/American Stroke Association (AHA/ASA) guidelines [2]. Of these, 34/41 (82.9%) stroke patients were documented by Magnetic Resonance Imaging (MRI) in the acute phase (Diffusion-Weighted Imaging-Alberta Stroke Program Early Computed Tomography Score (DWI-ASPECTS): 7 (7–8), stroke volume on Diffusion-Weighted Imaging (DWI): 9.8 (5.2–20.6) cm^3^). Further, 21/41 (51.2%) patients received intravenous alteplase prior to the endovascular procedure. At 24 h after the reperfusion treatment, asymptomatic hemorrhagic infarcts were observed on systematic brain imaging for 13/41 (31.7%) patients. At 3 months, 18/41 (43.9%) patients had a favorable clinical outcome defined by a mRS of 0 to 1 and 3/41 (7.3%) patients died by 3 months. The subtype etiology of stroke defined according to the Trial of Org 10,172 in Acute Stroke Treatment (TOAST) classification were distributed as follows: 32/41 (78.1%) cardioembolism, 2/41 (4.9%) large-artery atherosclerosis, 2/41 (4.9%) stroke other determined etiology and 5/41 (12.2%) stroke of undetermined etiology. As expected from the literature, age (*p* = 0.0247), baseline clinical gravity assessed by the NIHSS score (*p* = 0.0480) and admission glycemia (*p* = 0.0378) were significantly associated with clinical outcome (according the modified Rankin Scale (mRS)) in the univariate analysis presented in Table 1. In contrast, the medical history, radiological variables, thrombolytic use, timing of recanalization, hemorrhagic complications or even stroke etiological subgroups were not statistically associated with clinical prognosis (Table 1).

### 2.2. Composition of Cerebral Thrombi Assessed by Proteomic Analyses

We performed proteomic analyses to compare the composition of cerebral thrombi from stroke patients according to their clinical outcome. The cerebral thrombi were assessed by proteomic analysis for cell-type specific biomarkers [8]. Based on previously published immunohistochemical (IHC) data of cerebral thrombi, the following protein biomarkers were used in our study: red blood cells (RBC) (glycophorin-A), platelets (platelet glycoprotein Ib alpha chain, integrin beta-3, platelet endothelial cell adhesion molecule), leukocytes (receptor-type tyrosine-protein phosphatase C), fibrin (fibrinogens α, β and γ) and the von Willebrand factor. We did not detect any significant difference in the levels of these proteins in the cerebral thrombi. Thus, the cell-type composition was similar for all thrombi and, consequently, it was not related to the individual clinical outcome of the patients (Table 2).

### 2.3. Untargeted Metabolomic Analyses of Cerebral Thrombi

The metabolome of 41 cerebral thrombus samples retrieved by successful mechanical thrombectomy after large artery occlusion (mechanical Thrombectomy in Cerebral Infarction (mTICI) 2b/3) were compared according to the clinical outcome of the patients at 3 months after stroke. Post-treatment of the Liquid Chromatography-Mass Spectrometry (LC-MS) data revealed a total of 5019 molecular features (*m/z*) in both positive and negative electrospray ionization modes. A metabolomic data matrix is given in the Supplementary Materials.

**Figure 1:**
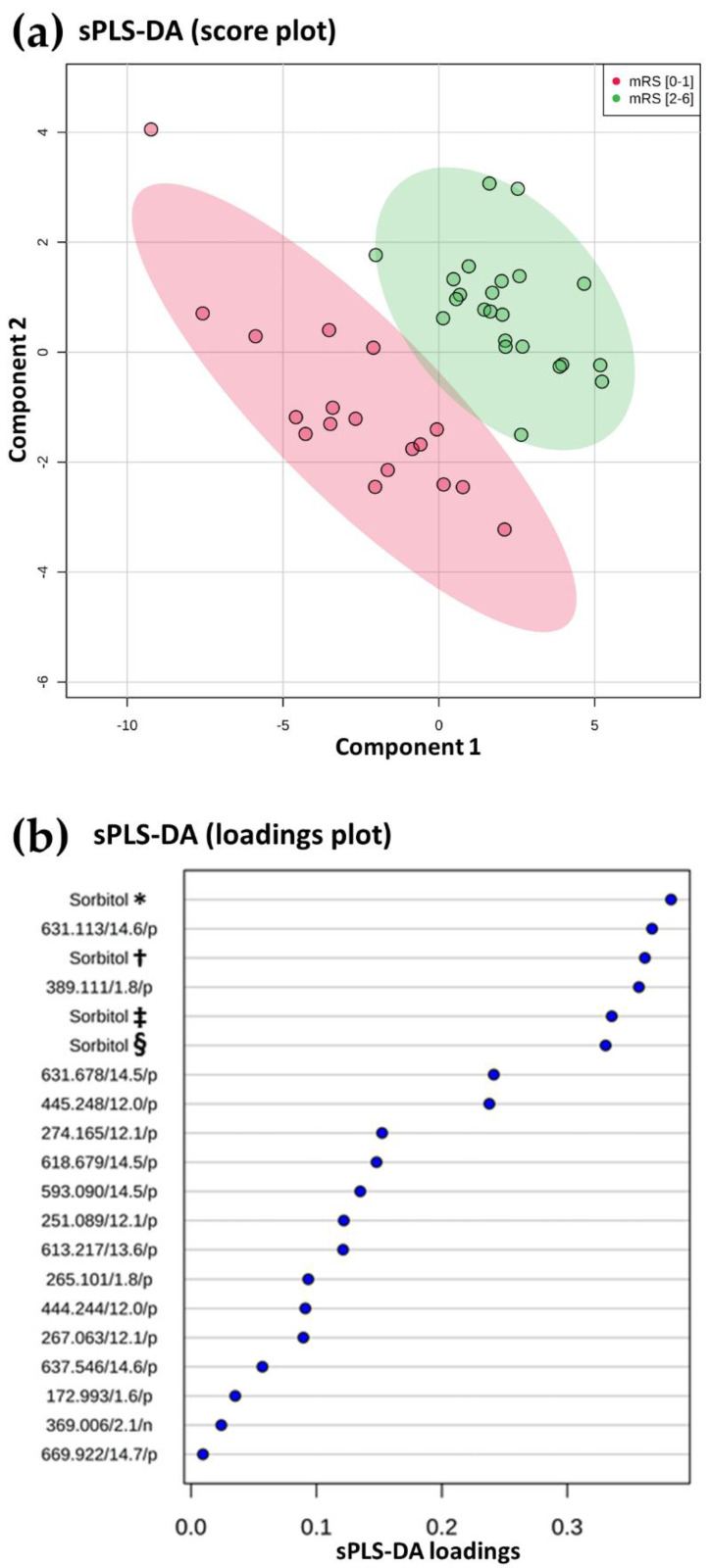
Metabolomic analysis of cerebral thrombi according to the clinical prognosis. sPLS-DA analysis based on untargeted metabolomic data of cerebral thrombi discriminated stroke patients according to their clinical outcomes (red and green dots represent respectively, patients with mRS (0–1) and mRS (2–6) at 3 months). The score plot is represented with a confidence ellipse of 95% (**a**). Loading plot of top 20 features selected on the first component of the sPLS-DA model. Identified features are shown as chemical names or at default by their mz/retention time (min)/ionization mode (*p*: positive and n: negative). Sorbitol was found in different adduct forms: * [M+H]^+^, † [M+H]^+^(^13^C_2_), ‡ [M+H-H_2_O]^+^ and § [M+H-H_2_O]^+^(^13^C) (**b**).

First, we determined if untargeted metabolomic analysis of cerebral thrombi was able to discriminate stroke clinical outcome. We therefore performed a supervised multivariate analysis (sPLS-DA) using MetaboAnalyst. As shown in Figure 1a, the metabolomes of cerebral thrombi discriminated between stroke patients with a favorable clinical outcome (mRS < 2) and stroke patients with poor outcome (mRS ≥ 2 at 3 months) (Area Under the Curve (AUC): 0.992 (0.931–1)). Metabolites selected by the sparse Partial Least Squares Discriminant Analysis (sPLS-DA) model were ranked according to their loadings (coefficients). Figure 1b shows the loading plot of the top 20 features selected on the first component of the sPLS-DA model. This selected feature revealed sorbitol adducts and isotopes as the main contributors ranked in the top 20 of the loading plots (Figure 1b). Interestingly, sorbitol [M+H]^+^ levels were significantly higher in cerebral thrombi from stroke patients with a favorable clinical outcome defined by a mRS (0–1) compared to other stroke patients (ion intensity, median (95% interquartile range (IQR)): 6.84 × 10^8^ (6.06 × 10^8^–7.90 × 10^8^) versus 5.24 × 10^8^ (4.52 × 10^8^–5.60 × 10^8^), *p* < 0.0001).

**Figure 2:**
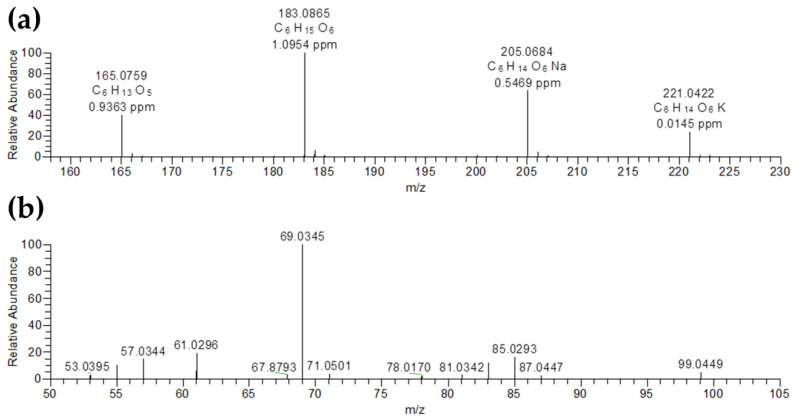
LC-MS/MS identification of sorbitol (C_6_H_14_O_6_; Metlin ID 147; [M]: 182.0790). Firstly, the following adducts of sorbitol were found at the same retention time (RT: 1.75 min) in positive ionization mode: [M+H]^+^ at *m/z* 183.0865, [M+H-H_2_O]^+^ at *m/z* 165.0759, [M+Na]^+^ at *m/z* 205.0684 and [M+K]^+^ at *m/z* 221.0422) (**a**). Secondly, a MS/MS fragmentation of [M+H]^+^ at *m/z* 183.0865 with a (+) 30V collision energy was performed (**b**).

The LC-MS identification of sorbitol (chemical formula: C_6_H1_4_O_6_; Metlin ID: 147; Monoisotopic mass [M]: 182.0790 Da) was confirmed as follows (Figure 2). Firstly, the following adducts of sorbitol were found at the same retention time (RT: 1.75 min) in the positive ionization mode: [M+H]^+^ ions at *m/z* 183.0865, [M+H-H_2_O]^+^ ions at *m/z* 165.0759, [M+Na]^+^ ions at *m/z* 205.0684 and [M+K]^+^ ions at *m/z* 221.0422 (Figure 2a). Secondly, MS/MS fragmentation of [M+H]^+^ ions at *m/z* 183.0865 with a +30 V collision energy (Figure 2b) matched with the spectral MS/MS fragmentation of the Metlin database (https://metlin.scripps.edu/metabo_info.php?molid=143) and MoNA–MassBank of North America (https://mona.fiehnlab.ucdavis.edu/spectra/display/RP022903).

### 2.4. Analyses of the Polyol (Sorbitol) Pathway in Cerebral Thrombi Cells Assessed by Metabolomic and Proteomic Analyses

**Figure 3:**
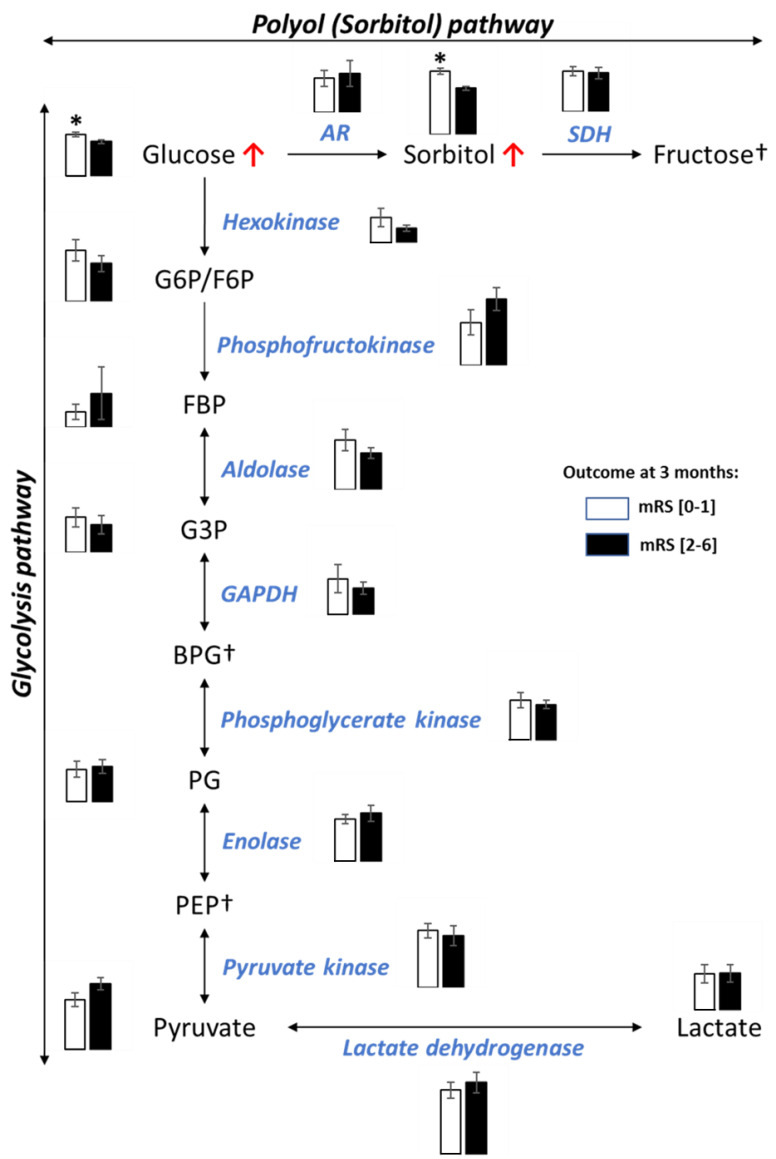
Metabolomic and proteomic analyses of the polyol (sorbitol) and glycolytic pathways of cerebral thrombi. A bar chart is shown for each identified metabolite and protein. White and black bars indicate the level of abundance in cerebral thrombi from patients respectively, with favorable and unfavorable outcomes. * *p* ≤ 0.05. † non-identified metabolite. G6P: glucose-6-phosphate; F6P: fructose-6-phosphate; FBP: fructose-1,6-biphosphate; G3P: glyceraldehyde-3-phosphate; BPG: biphosphoglycerate; PG: phosphoglycerate; PEP: phosphoenolpyruvate; AR: aldolase reductase, SDH: sorbitol dehydrogenase; GAPDH: glyceraldehyde-3-phosphate dehydrogenase.

The untargeted metabolomic analyses of cerebral thrombi indicated that sorbitol, an intracellular metabolite, is a main feature of the clinical prognosis for stroke. We therefore focused our study on the cellular pathways of this metabolite. Human erythrocytes are freely permeable to glucose, and insulin is not required for its cellular transport. Thus, the intracellular glucose levels in red blood cells (RBCs) vary directly with the plasmatic glucose concentration. Thereby, when plasmatic glucose is high, the RBC level of glucose is consequently high, and excess glucose is converted into sorbitol by the polyol (sorbitol) pathway [9]. Here, we performed an integrative metabolomic and proteomic analysis to compare the glycolytic and polyol pathways in cerebral thrombi according to the clinical outcome (Figure 3). We found that the level of glucose, the substrate of aldolase reductase sorbitol synthesis, was higher in cerebral thrombi of patients with favorable outcomes. However, this higher level of glucose in cerebral thrombi of stroke patients with a favorable outcome was not associated with modifications in the glycolytic pathway. In fact, the proteomic analysis did not show significant differences in levels of the enzymes of the glycolytic and polyol pathways in both subgroups of patients. In addition, the levels of the other detected metabolites of these pathways did not change significantly. Moreover, a linear correlation between glucose and sorbitol levels (R = 0.44; *p* = 0.0043) in cerebral thrombi was detected. This finding strongly suggests that the intracellular excess of glucose was not metabolized through glycolysis but converted into sorbitol by the polyol pathway.

In conclusion, these results indicate that a high level of sorbitol in cerebral thrombi is related to a high level of plasmatic glucose not metabolized by the glycolytic pathway in RBCs. Considering that sorbitol cannot cross the cell membrane, intracellular accumulation of sorbitol in cerebral thrombi from stroke patients with a favorable outcome should reflect a higher RBC and plasmatic level of glucose at stroke onset.

### 2.5. Predictive Value Comparison between Sorbitol in Cerebral Thrombi Reflecting Glycemia at Stroke Onset and Admission Glycemia

Admission hyperglycemia was significantly associated with an unfavorable clinical outcome in stroke patients [4,5]. Our metabolomic results indicated that the high intracellular level of glucose of RBCs at stroke onset (revealed by high sorbitol level in cerebral thrombi) was associated with a favorable clinical outcome. The independent property of the two prognostic features were subsequently assessed using a multivariate analysis. In the logistic regression model, we included the following variables significantly associated with favorable outcome in previous univariate analyses: sorbitol, admission glycemia, age and baseline NIHSS. Sorbitol in cerebral thrombi was the only significant independent predictor of favorable clinical prognosis for stroke patients successfully recanalized after MCA occlusion (odds ratio (OR) for sorbitol (per 10^7^ arbitrary unit): 1.24 (1.07–1.44); *p* = 0.004).

**Figure 4:**
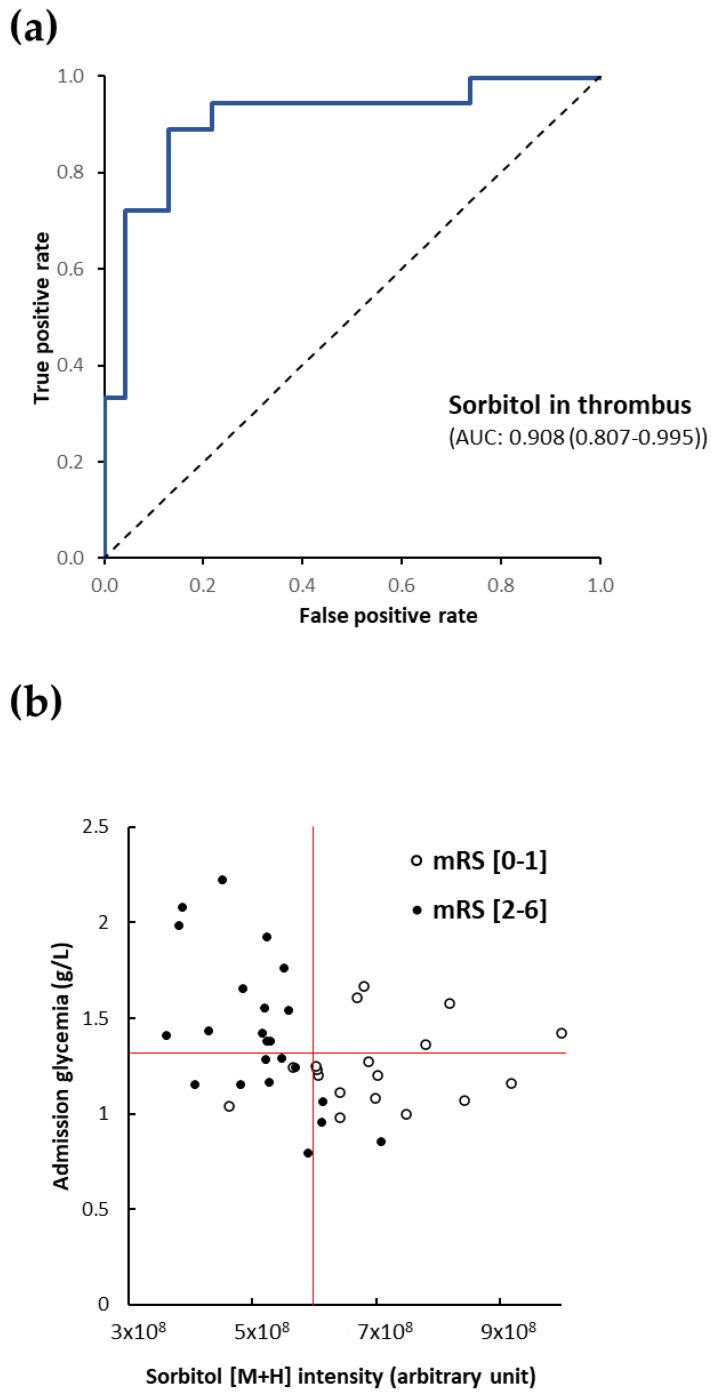
Sorbitol in cerebral thrombi and admission glycemia in the prediction of the clinical prognosis (mRS) for stroke patients. ROC curve of sorbitol in cerebral thrombi for predicting favorable outcome (**a**). Scatter plot showing correlation between the level of sorbitol [M+H]^+^ in cerebral thrombi and admission glycemia according to the clinical outcome. Red lines represent the best thresholds (Youden plots) of each predictor of favorable outcome (sorbitol in cerebral thrombi ≥ 5.9 × 10^8^ and admission glycemia < 1.27 g/L) (**b**).

To test the predictive values, areas under the ROC curve (AUC) for sorbitol were obtained (Figure 4a). The AUC value of sorbitol to predict clinical favorable outcome was 0.908 (0.807–0.995). For comparison, the AUC value of admission glycemia to predict poor outcome was 0.644 (0.470–0.818). Using the optimal threshold at Youden plot (sorbitol [M+H]^+^ ion intensity > 5.90 × 10^8^), sensibility, specificity, positive and negative predictive values of sorbitol were respectively 88.89%, 86.96%, 84.2% and 90.9%.

Our findings illustrated in Figure 4b by a scatter plot show correlation between the level of sorbitol [M+H]^+^ in cerebral thrombi and admission glycemia according to the clinical outcome. Red lines separate the patients according to optimal decision thresholds of the level of sorbitol in cerebral thrombi (vertical line) and admission glycemia (horizontal line) calculated from the ROC curves. Indeed, the scatter plot shows the significant relationship between favorable clinical prognosis (open circles) and high sorbitol levels in cerebral thrombi (on the right of the vertical red line) independent of the glycemia level at admission. Interestingly, among the stroke patients with low sorbitol levels (on the left of the vertical red line) in cerebral thrombi, 13/22 (59.1%) patients had high glycemia (upper of the horizontal red line) at admission, thus combining both predictors of poor outcome, and all these patients (100%) showed an unfavorable outcome. Only nine patients had low sorbitol levels in cerebral thrombi and low glycemia at admission and two of them showed a favorable outcome. These very low numbers of patients are too low to evaluate if admission glycemia predicts the outcome of patient with low sorbitol (glycemia) at stroke onset. Additional clinical studies including analyses of the sorbitol levels in cerebral thrombi are required. These observations indicate that the sorbitol level in the cerebral thrombus is the only independent and efficient clinical outcome predictor.

**Figure 5:**
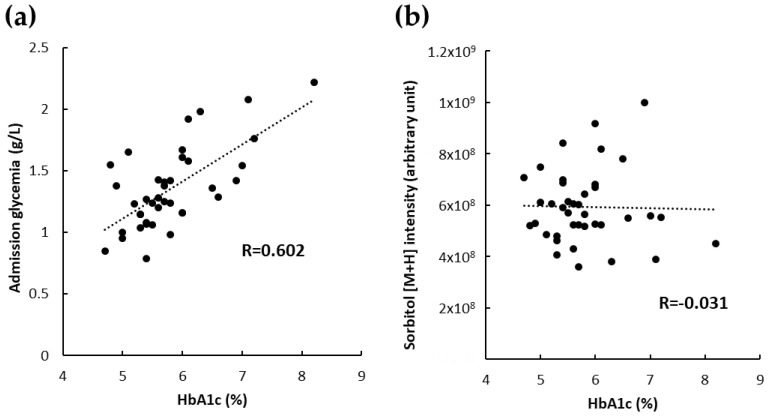
Correlations between glycated hemoglobin (HbA1c), sorbitol in cerebral thrombi and admission glycemia. HbA1c was positively correlated with admission glycemia (**a**) but not with sorbitol in cerebral thrombi (**b**).

We also performed a correlation analysis between admission glycemia, the sorbitol level in cerebral thrombi and glycated hemoglobin (HbA1c), a marker of long-term glycemic control. Interestingly, we found a significant linear correlation between admission glycemia and HbA1c (R = 0.602; *p* < 0.001) (Figure 5a). In contrast, the sorbitol levels were independent of the HbA1c levels (R = −0.031; *p* = 0.853) (Figure 5b).

## 3. Discussion

The results of untargeted metabolomic analyses of cerebral thrombi revealed the importance of glycemia at stroke onset for the clinical outcome of patients with large vessel occlusion successfully recanalized by mechanical thrombectomy within 6 h of symptom onset. Our findings highlight the beneficial effect of glucose at stroke onset on the clinical outcome contrasting with the unfavorable outcome related to hyperglycemia at admission [4,5]. Our study also showed that metabolomic analyses of cerebral thrombi is an efficient tool for the prediction of the clinical outcome. Furthermore, our results indicate that a high sorbitol level in cerebral thrombi, reflecting a higher glycemia level at stroke onset, was strongly associated with favorable clinical outcome.

Our integrative proteomic and metabolomic analysis permitted the comparison of the levels of enzymes and metabolites of the glycolytic and polyol pathways in cerebral thrombi according to the clinical outcome in large vessel occlusion stroke successfully recanalized by mechanical thrombectomy within 6 h of symptom onset. The proteomic analysis showed no significant difference in the levels of protein biomarkers of each thrombus, suggesting that their composition was homogeneous for the two clinical outcome groups. In addition, the proteomic analysis indicated no difference in the levels of enzymes of the glycolytic and polyol pathways. Aldolase reductase, the first-step reaction of the polyol (sorbitol) pathway, converts intracellular glucose into sorbitol [10]. The polyol pathway is present in several cell types including RBCs, in which it has been particularly studied. RBC sorbitol was used in the past as an indicator of short-term glycemic control [9,10,11,12]. The metabolomic analysis showed that sorbitol and glucose were in higher abundance in cerebral thrombi from stroke patients with a favorable outcome. The higher level of glucose in cerebral thrombi was not associated with a change in glycolytic flux (shown by proteomic and metabolomic results) and was positively correlated with sorbitol. Therefore, and in agreement with the literature, we propose that the higher level of sorbitol in cells, and particularly in the RBCs of cerebral thrombi, was the consequence of an excess of plasmatic glucose not metabolized through the glycolytic pathway [9,10,11,12] at stroke onset. Indeed, one should note that the RBC plasma membrane has a high insulin-independent glucose transport activity and glucose should easily diffuse from the extracellular space. The intracellular glucose and sorbitol levels vary directly with the plasmatic glucose concentration [9,10,11,12]. The sorbitol level in RBC, the product of the polyol pathway (a sub-pathway of glycolysis), logically correlated to variations in the plasmatic concentration of glucose. However, sorbitol is an intracellular metabolite that does not spontaneously diffuse across cell membranes. Therefore, intracellular accumulation of sorbitol in RBCs is induced by hyperglycemia, which is in agreement with previous reports [9,10,11,12]. Despite a limited number of patients, these results clearly indicate that the level of glycemia at stroke onset is a strong indicator of favorable clinical outcome at 3 months (mRS < 2) in stroke patients with large vessel occlusion successfully recanalized by mechanical thrombectomy within 6 h of symptom onset. Our results show that high plasmatic glucose at stroke onset, assessed by the sorbitol level in RBCs of cerebral thrombi, could prevent injury in ischemic tissues, thereby leading to a favorable clinical outcome. As the most important fuel for the brain, the beneficial role of glucose may stem from its ability to fuel the energy demands in the hypoperfused tissue by anaerobic glycolysis under ischemic conditions after stroke onset. Because the consumption of glucose is slowed down in the hypoperfused region by cessation of electrical activity [6], anaerobic glycolysis could theoretically supply sufficient ATP to the penumbra to maintain cell viability until reperfusion, thereby preventing the ischemic cascade [6]. However, glucose consumption could also induce deleterious effects, the cost of ATP production by anaerobic glycolysis is lactic acid accumulation and tissue acidosis, proportional to the blood glucose level [13,14]. Tissue acidosis has been considered as the main cause of the deleterious effect of glucose, known as the “glucose paradox” of stroke [6,15,16]. However, the underlying mechanisms of the deleterious effect targeting directly tissue injury including the “lactate acidosis hypothesis” are still under debate [6,15,16]. Interestingly, it was reported that the slight decrease in pH in ischemic lesions under hyperglycemic conditions was not sufficient to exacerbate stroke injury [6]. Accordingly, our findings here showed that the higher plasmatic level of glucose at stroke onset, assessed by a higher sorbitol level in RBC of cerebral thrombi, induced a beneficial clinical effect in stroke patients with middle cerebral artery occlusion completely recanalized within 6 h.

For the patients included in the studied cohort, high levels of plasmatic glucose on admission (≥1.27 g/L) was weakly but significantly associated with an unfavorable outcome. This result is in agreement with several clinical studies [4,5] and indicates that our cohort is reproducible for patients with large vessel occlusion successfully recanalized by mechanical thrombectomy. Admission hyperglycemia has been associated with a poor functional outcome in patients treated with intravenous rt-PA and more recently in stroke patients with large vessel occlusion treated with mechanical thrombectomy [4,5]. Admission hyperglycemia is common in acute ischemic stroke and could be caused by a stress response to acute neurologic injury or poor control of blood glucose levels in patients with diabetes mellitus [6,7]. It was not surprising to find that admission glycemia strongly correlated to the HbA1c level in our study. HbA1c should be considered as a long-term indicator of glycemic control, indicating as expected that hyperglycemia did not occur just before admission. In contrast, the sorbitol level in RBCs of cerebral thrombi should be considered as a short-term indicator of hyperglycemia. However, as discussed above, the sorbitol level in cerebral thrombi is related to glycemia at stroke onset and, therefore, as expected, did not correlate with the level of glycemia of patients on admission. The limited number of patients of this study does not allow us to conclude if, for patients with a low level of glycemia at stroke onset, an unfavorable clinical outcome is related to admission hyperglycemia. In contrast, our results show that, for patients with hyperglycemia at stroke onset (related to favorable outcome), admission hyperglycemia had no significant deleterious effect.

Moreover, the measurement of blood glucose at admission also provided information about glycemia just before reperfusion. In agreement with a deleterious role of glucose in the reperfusion setting, clinical studies indicate a negative effect of admission hyperglycemia linked to recanalization. Alvarez-Sabin et al. [17], and more recently Rosso et al. [18], showed that the detrimental effect of acute hyperglycemia in stroke patients was higher after early reperfusion, compared to delayed or no reperfusion. Despite the heterogeneity of the animal models studied, experimental studies reveal a deleterious effect of hyperglycemia in the setting of reperfusion [6,15], while little or no detrimental effect of hyperglycemia was shown in complete ischemia [19]. Here again, the limited number of patients of this study does not allow us to conclude if, for patients with a low level of glycemia at stroke onset, an unfavorable clinical outcome is related to hyperglycemia during reperfusion. In contrast, hyperglycemia at stroke onset appears to have a dominant protective effect on hyperglycemia during reperfusion. This could explain why insulin targeting of hyperglycemia on admission aimed at stroke neuroprotection does not provide significant clinical benefit [20]. Strategies of neuroprotection targeting glucose regulation must be reassessed in the light of our results.

At the cellular level, the opposing effects of glucose could differ with the metabolic heterogeneity of cells between the core and the penumbra area [6]. In the ischemic core, the cerebral tissue is poorly perfused or not perfused at all. We would expect that only the entrapped glucose at stroke onset, determined by the plasmatic level at this time, could be metabolized in the ischemic core. As discussed above, in the penumbra region, the beneficial effect of hyperglycemia may be canceled out by lactic acidosis due to the continuous supply of glucose by the collateral circulation in the context of oxygen restriction. According to our results showing the dominant effect on favorable outcome of hyperglycemia at stroke onset, we could hypothesize that the related beneficial effects are aimed mainly at the core area or area with a very restricted collateral circulation. This beneficial effect may be explained by the ability of glucose to supply the ATP demand without exacerbating deleterious acidosis [6]. Interestingly, it was in settings of poor collateral circulation, such as lacunar stroke affecting deep matter regions, end-arterial vascular territories stroke or in the core of large cortical ischemic stroke, in which hyperglycemia was not associated with a deleterious effect but potentially beneficial [6]. In addition, one should note that injuries of end-arterial vascular territories such as deep hemispheric white matter are associated with poor clinical outcome in proximal middle cerebral artery occlusion stroke [21]. Therefore, we propose that a higher glycemic level at stroke onset could be associated with the rescue of this clinically relevant tissue after recanalization.

## 4. Materials and Methods

### 4.1. Patient Selection and Procedure

Our ThrombiOMIC database included metabolomic and proteomic analyses of cerebral thrombi successfully retrieved from all consecutive stroke patients with large vessel occlusion treated by mechanical thrombectomy in an acute phase between October 2018 and August 2019 in the intensive care stroke unit of the University Hospital of Nice (France). In the ThrombiOMIC database, each stroke patient was clinically assessed by a neurologist at baseline and treated according to AHA/ASA guidelines [2]. We collected pre-therapeutic radiological data such as the DWI stroke volume on MRI, Alberta Stroke Program Early Computed Tomography Score (DWI-ASPECTS) and the occlusion site using the 3D Time-Of-Flight (3D-TOF MRA) and/or CT angiography. Reperfusion treatment included thrombolysis by alteplase if eligible before the thrombectomy. At the end of the endovascular procedure, the modified Thrombolysis in Cerebral Infarction scale (mTICI) was evaluated by a neuroradiologist. Brain imaging (CT or MRI) was performed systematically 24 h after mechanical treatment. Intracranial hemorrhage (ICH) was assessed according to ECASS2 (European Collaborative Acute Stroke Study) classification by a neuroradiologist. At 3 months, the Modified Rankin Scale (mRS) was performed during a clinical follow-up consultation by a certified neurologist. A mRS of 0 to 1 was considered as a favorable outcome. Strokes were classified into etiological subtypes using the TOAST classification.

In this study, we included, from the ThrombiOMIC database, stroke patients (National Institutes of Health Stroke Scale (NIHSS) score ≥ 6 and Alberta Stroke Program Early CT score (ASPECT) ≥6) with occlusion of middle cerebral artery (MCA M1) or tandem occlusion (MCA M1/ICA) completely recanalized by mechanical thrombectomy (defined by mTICI scale 2a or 3) within 6 h from stroke onset [2]. Stroke patients with parenchymal hematoma after reperfusion treatment were excluded from this study. Cerebral thrombi were collected for routine practice. Therefore, our study was carried out following the rules of the Declaration of Helsinki. Ethics approval and patient agreement was not required for this non-interventional study.

### 4.2. Collection and Processing of Cerebral Thrombi for Omic Analyses

Each thrombus, in single or multiple fragments, was washed with cold saline solution in situ and stored at −80 °C. Initial processing of cerebral thrombi was the same for the proteomic and metabolomic analyses. Cerebral thrombi were rinsed first in water and then in a protease/phosphatase inhibitor solution at 4 °C (cOmplete, EDTA-free and PhosSTOP; Roche Diagnostics GmbH, Germany). Thrombectomy material was manually homogenized in lysis buffer (7 M urea, 2 M thiourea, 4% DDM, 50 mM DTT) and incubated under agitation at 4 °C for 20 min. After centrifugation (13,000× *g* for 15 min at 4 °C), supernatants were collected and protein concentrations were determined by the Bradford assay (Bio-Rad Protein Assay; Bio-Rad, Marnes-la-Coquette, France). For metabolomic analyses, volumes of supernatant corresponding to 80 µg of protein were mixed with five volumes of methanol (HPLC grade, Merck Millipore, Molsheim, France) and incubated overnight at −20 °C for protein precipitation. After centrifugation, supernatants was removed, dried using a SpeedVAC concentrator (SVC100H, SAVANT, Thermo Fisher Scientific, Illkirch, France), resuspended in 80 µL of a 20:80 acetonitrile-H_2_O mixture (HPLC grade, Merck Millipore) and stored at −20 °C until use for metabolomic analysis. For proteomic analyses, volumes of supernatant corresponding to 100 µg of protein were loaded onto a urea polyacrylamide gel. After penetration of the proteins into the gel, electrophoresis was stopped, and protein-containing gel portions were recovered. Then, proteolytic in-gel digestion with trypsin was performed. After centrifugation, the peptide-containing supernatant was dried and resuspended in 50 µL of a 20:80 acetonitrile-H_2_O mixture and stored at −20 °C until use.

### 4.3. Metabolomic Analyses

Chromatographic analysis was performed with the DIONEX Ultimate 3000 HPLC system coupled to a chromatographic column (Phenomenex Synergi 4 u Hydro-RP 80A 250_3.0 mm) set at 40 °C and a flow rate of 0.9 mL/min. Gradients of mobile phases (mobile phase A: 0.1% formic acid in water and mobile phase B: 0.1% formic acid in acetonitrile) were performed over a total of 25 min. MS analysis was carried out on a Thermo Scientific Exactive Plus Benchtop Orbitrap mass spectrometer. The heated electrospray ionization source (HESI II) was used in positive and negative ion modes. The instrument was operated in full scan mode from *m/z* 67 to *m/z* 1000. Post-treatment of data was performed using the MZmine2 version 2.53 (http://mzmine.github.io/) [22]. Metabolites were identified using the Metlin database (https://metlin.scripps.edu/).

### 4.4. Proteomic Analysis

The obtained peptides (5 μL) were analyzed using an ESI-Q Exactive Plus mass spectrometer incorporating a high-field Orbitrap analyzer coupled to an Ultimate 3000 RSL C Nano LC System (Thermo Fisher Scientific). For nano-liquid chromatography, the system was set up in a pre-concentration mode using a 300 μm × 5 mm trap column in back-flush configuration at 40 °C (*p*/N 6720.0315). An EASY-Spray 25 cm × 75 μm and 2 μm diameter *p*/N ES 802A) was connected and coupled to the system with an EASY-Spray source (*p*/N ES081) operating at 40 °C. The flow rate was 0.3 μL/min with a 5–45% gradient of solvent B (80% acetonitrile, 20% water, 0.1% formic acid) against solvent A (0.1% formic acid, 100% water) for 120 min. Full-scan mass spectra were measured from 350 to 1500 *m/z* with an Automatic Gain Control Target. All MS raw data files were analyzed by Proteome Discoverer software 1.4 (Thermo Fisher, France) using the Sequest HT search engine against a database of protein sequences (Uniprot version2015_2).

### 4.5. Statistical Analysis

Pre-therapeutic continuous variables are presented as the mean with standard deviation or the median with the interquartile range. Categorical variables are presented by absolute numbers (%). Statistical association between variables and clinical outcomes was assessed in a univariate analysis using the Chi^2^ test for categorical variables and the Student’s *t* test or Wilcoxon-Mann-Whitney test for continuous variables. The correlation analyzes were performed using the Spearman’s rank correlation test. *p* < 0.05 was considered significant. Statistical analyses were conducted using the STATA 10.0.

Statistical analyses of the untargeted metabolomic analyses of thrombi were processed using statistical modules proposed by MetaboAnalyst 4.0 (https://www.metaboanalyst.ca) [23]. The statistical analysis module was used. No pre-processing data was performed before the statistical analysis including data filtering, sample normalization, data transformation, and data scaling. Using sparse Partial Least Squares-Discriminant Analysis (sPLS-DA), we analyzed loadings (coefficients) of features selected by the sPLS-DA model (variables per component: 20; number of components: 2) to identify important metabolites associated with a favorable clinical outcome. Classical statistical tests to measure association between major metabolites and the clinical outcome such as the Receiver-Operating Characteristic (ROC) curve and Youden plot were performed using the biomarker analysis module. The ROC curve of the sPLS-DA model was constructed from the top 20 features selected for each component (40 variables).

## 5. Conclusions

This study highlights the dominant and beneficial clinical effect of glucose assessed at stroke onset and provides new insight into the opposing effects of an increase in glycemia on stroke and before reperfusion. Metabolomic analysis of cerebral thrombi showed that a high sorbitol level reflecting glycemia at stroke onset is an independent predictor of favorable clinical outcome in large vessel occlusion stroke patients successfully recanalized by thrombectomy. Metabolomic analysis of cerebral thrombi is a new tool for glucose evaluation at stroke onset. Our approaches offer new perspectives for comprehension of the opposing roles of glucose in stroke [6] and for the development of new neuroprotection strategies at the acute phase of stroke targeting metabolism and energetics of the brain during pre-hospital management.

## Figures and Tables

**Table 1:** Baseline characteristics of stroke patients. * *p <* 0.05.

Patient Characteristics		All Patients (*n* = 41)	mRS < 2 (*n* = 18)	mRS ≥ 2 (*n* = 23)	*p*
**Demographic characteristics**					
Age, years	*	74.8 ± 13.2	70.2 ± 16.7	78.3 ± 12.7	0.0247
Gender, male		17 (41.5%)	5 (27.8%)	12 (52.2%)	0.2010
**Medical history**					
Hypertension		17 (41.5%)	6 (33.3%)	11 (47.8%)	0.5240
Diabetes mellitus		4 (9.8%)	1 (5.6%)	3 (13.0%)	0.6180
Coronary artery disease		4 (9.8%)	3 (16.7%)	1 (4.4%)	0.3030
Smoking		6 (14.6%)	4 (22.2%)	2 (8.7%)	0.3770
Atrial fibrillation		29 (70.7%)	12 (66.7%)	17 (73.9%)	0.7340
**Clinical characteristics**					
Baseline NIHSS score	*	17 (15–22)	15 (13–21)	20 (16–22)	0.0480
Baseline blood glucose, mg/dL	*	134 ± 32	125 ± 21	142 ± 38	0.0378
**Imaging characteristics**					
Tandem ICA/MCA occlusion		5 (12.2%)	1 (5.6%)	4 (17.4%)	0.3630
MRI brain imaging		34 (82.9%)	15 (83.3%)	19 (82.6%)	1.0000
DWI-ASPECTS		7 (7–8)	8 (7–8)	7 (6–7)	0.1180
DWI-Volume, cm^3^		9.8 (5.2–20.6)	8.8 (4.9–16.6)	12.6 (5.8–27.5)	0.2746
**Treatment characteristics**					
Intravenous thrombolysis		21 (51.2%)	10 (55.6%)	11 (47.8%)	0.7560
Time to recanalization, min		252.4 ± 62.3	245.8 ± 49.4	257.5 ± 71.4	0.2689

**Table 2:** Composition of cerebral thrombi from stroke patients determined by proteomic analysis depending on their clinical outcomes.

Components	Description	mRS (0–1)	mRS (2–6)	*p* Value
RBC	Glycophorin-A	1.67 × 10^8^ ± 2.70 × 10^7^	1.35 × 10^8^ ± 1.69 × 10^7^	0.3345
Platelets	Platelet glycoprotein Ib alpha-chain	4.63 × 10^7^ ± 1.12 × 10^7^	4.66 × 10^7^ ± 1.35 × 10^7^	0.9884
	Integrin beta-3	9.81 × 10^8^ ± 1.48 × 10^8^	9.46 × 10^8^ ± 1.85 × 10^8^	0.8881
	Platelet endothelial cell adhesion molecule	1.70 × 10^8^ ± 2.76 × 10^7^	1.73 × 10^8^ ± 3.90 × 10^7^	0.9510
Leukocytes	Receptor-type tyrosine-protein phosphatase C	2.00 × 10^7^ ± 3.66 × 10^6^	2.31 × 10^7^ ± 4.74 × 10^6^	0.6281
Fibrin	Fibrinogen alpha chain	5.49 × 10^8^ ± 1.78 × 10^8^	1.38 × 10^9^ ± 4.32 × 10^8^	0.1125
	Fibrinogen beta chain	5.01 × 10^9^ ± 1.18 × 10^9^	5.41 × 10^9^ ± 9.19 × 10^8^	0.7889
	Fibrinogen gamma chain	6.43 × 10^9^ ± 1.66 × 10^9^	8.38 × 10^9^ ± 1.49 × 10^9^	0.3880
von Willebrand factor	von Willebrand factor	5.94 × 10^7^ ± 1.96 × 10^7^	6.11 × 10^7^ ± 1.48 × 10^7^	0.9444

## Supplementary Materials

The following are available online at https://www.mdpi.com/2218-1989/10/12/483/s1, Metabolomic data matrix of 41 cerebral thrombus samples studied.
